# Eutopic and Ectopic Endometrial Interleukin-17 and Interleukin-17 Receptor Expression at the Endometrial—Myometrial Interface in Women with Adenomyosis: Possible Pathophysiology Implications

**DOI:** 10.3390/ijms252011155

**Published:** 2024-10-17

**Authors:** Le-Tien Hsu, Pei-Chen Lu, Yi-Wen Wang, Hsien-Ming Wu, I-Ju Chen, Hong-Yuan Huang

**Affiliations:** 1 Department of Obstetrics and Gynecology, Linkou Medical Center, Chang Gung Memorial Hospital, Taoyuan City 33305, Taiwan; letenhsu@gmail.com (L.-T.H.); t2003456@gmail.com (P.-C.L.); iwen0711@gmail.com (Y.-W.W.); danielwu@cgmh.org.tw (H.-M.W.); 2 Department of Family Medicine, Linkou Medical Center, Chang Gung Memorial Hospital, Taoyuan City 33305, Taiwan; b9602054@cgmh.org.tw; 3 Department of Obstetrics and Gynecology, College of Medicine, Chang Gung University, Taoyuan City 33305, Taiwan

**Keywords:** interleukin-17, adenomyosis, endometrial–myometrial interface, cytokine

## Abstract

Adenomyosis involves the infiltration of endometrial glands and stroma deep into the uterine tissue, causing disruption to the endometrial–myometrial interface (EMI). The role of interleukin-17 (IL-17) has been extensively studied in endometriosis, but its involvement in adenomyosis remains unclear. This study aimed to investigate the expression of IL-17 in eutopic and ectopic endometrium (adenomyosis) of individuals with adenomyosis at the level of EMI. Paired tissues of eutopic endometrium and adenomyoma were collected from 16 premenopausal women undergoing hysterectomy due to adenomyosis. The IL-17 system was demonstrated in paired tissue samples at the level of EMI by the immunochemistry study. Gene expression levels of IL-17A and IL-17 receptor (IL-17R) were assessed through quantitative real-time reverse transcription polymerase chain reaction (RT-PCR). Comparative gene transcript amounts were calculated using the delta-delta Ct method. By immunohistochemical staining, CD4, IL-17A, and IL-17R proteins were detected in both eutopic endometrium and adenomyosis at the level of EMI. IL-17A and IL-17R were expressed mainly in the glandular cells, and the expression of both IL-17A and IL-17R was found to be stronger in adenomyosis than in endometrium. 3-Diaminobenzidine (DAB) staining revealed greater IL-17A expression in adenomyosis compared to eutopic endometrium. Quantitative RT-PCR showed 7.28-fold change of IL-17A and 1.99-fold change of IL-17R, and the fold change level of both IL-17A and IL-17R is significantly higher in adenomyosis (IL-17A: *p* = 0.047, IL-17R: *p* = 0.027) versus eutopic endometrium. We found significantly higher IL-17 levels in adenomyosis compared to eutopic endometrium at the level of EMI. The results showed that the IL-17 system may play a role in adenomyosis.

## 1. Introduction

Adenomyosis is a condition where endometrial glands and stroma infiltrate the myometrium, leading to thickening of the surrounding muscle tissue. Patients with symptomatic adenomyosis often experience an enlarged uterus, abnormal uterine bleeding, and painful menstrual cycles. It can also occur alongside uterine leiomyoma and/or endometriosis. The epidemiology of adenomyosis remains unclear, as much of the available data have been based on examining the uterus after hysterectomy. Studies indicate that adenomyosis is found in a broad range of individuals undergoing hysterectomy, with reported rates varying from 9–62% [1].

Adenomyosis is a pathological condition of the uterus closely associated with endometriosis [2]. Similar to endometriosis, adenomyosis is reliant on estrogen and involves the ectopic presence of endometrial cells and stromal fibroblasts within the myometrium, either as extensions of the normally located endometrium or as islands surrounded by hypertrophic smooth muscle cells [3,4,5]. Adenomyosis shares similar symptoms with endometriosis, including heavy menstrual bleeding, dysmenorrhea, chronic pelvic pain, and infertility [5,6]. Several theories have been proposed to explain the pathogenesis of adenomyosis [7]. The prevailing understanding implicates the profound invasion of endometrial tissue into the inner myometrium, causing disruption to the endometrial–myometrial interface (EMI) [8,9]. Therefore, a series of molecular and metabolic disturbances occur in the eutopic and ectopic endometrium of women with adenomyosis. These disturbances stimulate angiogenesis and proliferation, impair apoptosis, produce estrogens, increase progesterone resistance, and downregulate cytokine expression. The endometrial tissue infiltrates the junctional zone myometrium, followed by the growth of ectopic endometrial glands [7,10]. Another prominent theory has been proposed to explain the origin of adenomyosis. It suggests that the condition arises from tissue injury and the subsequent repair process, leading to the invagination of the endometrial basalis into the myometrium [3,11].

Multiple lines of evidence suggest that inflammation and immune responses play a pivotal role in the pathogenesis of endometriosis [12,13]. In the same way, growing evidence indicates alterations in humoral and cellular immunity in adenomyosis [14,15]. Moreover, various immune-related serum proteins have been suggested to contribute to the development of adenomyosis [16]. A recent study suggested that IL-17 may play a crucial role in the pathogenesis of early endometriosis and endometriosis-associated infertility [17]. In addition, Hirata et al. first showed the presence of Th17 cells in the peritoneal fluid (PF) of endometriotic women by flow cytometry and the presence of interleukin-17A (IL-17A)-positive cells in endometriotic tissues by immunohistochemistry [18].

Interleukin-17 (IL-17) is secreted by the CD4+ T helper 17 (Th17) cell and is recognized as the hallmark cytokine of the unique cell population. In 1999, IL-17 was first identified through the use of T-cell clones derived from the joints of patients suffering from rheumatoid arthritis [19,20,21,22]. Currently, the IL-17 family comprises six structurally associated cytokines (IL-17A, IL-17B, IL-17C, IL-17D, IL-17E, and IL-17F), all sharing genetic homology, along with five matching receptors (IL-17RA (or IL-17R), IL-17RB, IL-17RC, IL-17RD, and IL-17RE) found on cell surfaces. IL-17A is the inaugural member of this family and holds the distinction of being the most extensively studied, previously thought to be predominantly generated by Th17 cells [23,24,25]. IL-17F and IL-17A demonstrate elevated genetic homology and have the capability to create both homodimers and heterodimers for signaling purposes. Moreover, they engage with the same receptor complex, thereby predominantly sharing biological functions, with IL-17A exhibiting greater potency compared to IL-17F. The four other IL-17 isoforms exist exclusively as homodimers [21,26,27,28].

So far, scant information exists concerning the role of the IL-17 system in adenomyosis, and there is no report regarding the presence of IL-17 in women with adenomyosis. To advance the understanding of the pathology and treatment of adenomyosis, this study aimed to investigate the expression of IL-17 in both ectopic and eutopic endometrium and to compare its expression between these two types of endometrium at the level of EMI in individuals with adenomyosis.

## 2. Results

**Figure 1:**
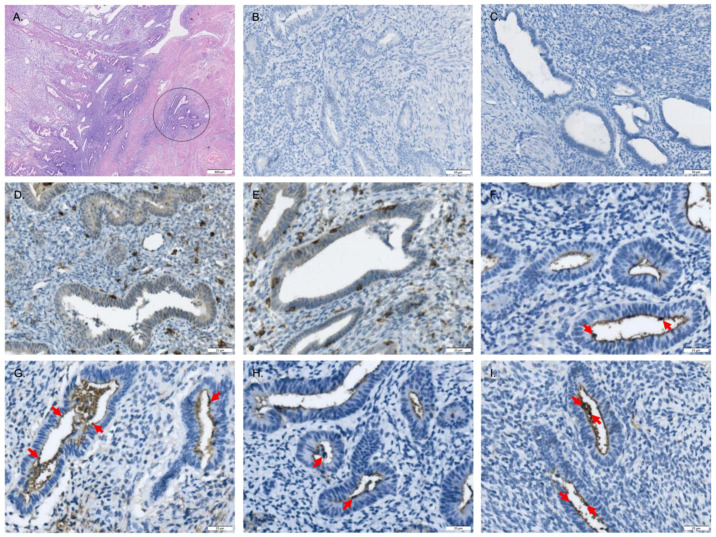
Immunohistochemistry of the IL-17 system in patients with adenomyosis. (**A**) Endometrial–myometrial interface stained by H&E, and the circled area indicates the adenomyotic lesion. (**B**,**C**) Negative controls without staining: (**B**) endometrium and (**C**) adenomyosis. The brown stain indicated infiltration of CD4-positive immune cells in both glandular and stromal tissues of endometrium (**D**) and adenomyosis (**E**). The brown stain (red arrow) showed the IL-17A positive reaction mainly in the glandular cells of endometrium (**F**) and adenomyosis (**G**), and the dyeing is denser in adenomyosis (**G**) than in endometrium (**F**). The brown stain (red arrow) showed the IL-17R positive reaction mainly in the glandular cells of endometrium (**H**) and adenomyosis (**I**), and the dye is denser in adenomyosis (**I**) than in endometrium (**H**). (**A**) scale bar = 400 μm, (**B**,**C**) scale bar = 50 μm, (**D**–**I**) scale bar = 25 μm. H&E: hematoxylin and eosin.

In Figure 1, the tissues from both eutopic and ectopic endometrium (adenomyosis), fixed in formalin, underwent preparation for immunohistochemical staining to reveal the existence of the IL-17 system and CD4 proteins at the EMI level. CD4, IL-17A, and IL-17R immunoreactive proteins were detected within both the eutopic endometrium and adenomyosis. Infiltration of CD4-positive immune cells was noted in both glandular and stromal tissues of the eutopic endometrium and adenomyosis. IL-17A and IL-17R were expressed mainly in the glandular cells of the eutopic endometrium and adenomyosis, and the expression of both IL-17A and IL-17R was found to be stronger in adenomyosis than in endometrium.

**Figure 2:**
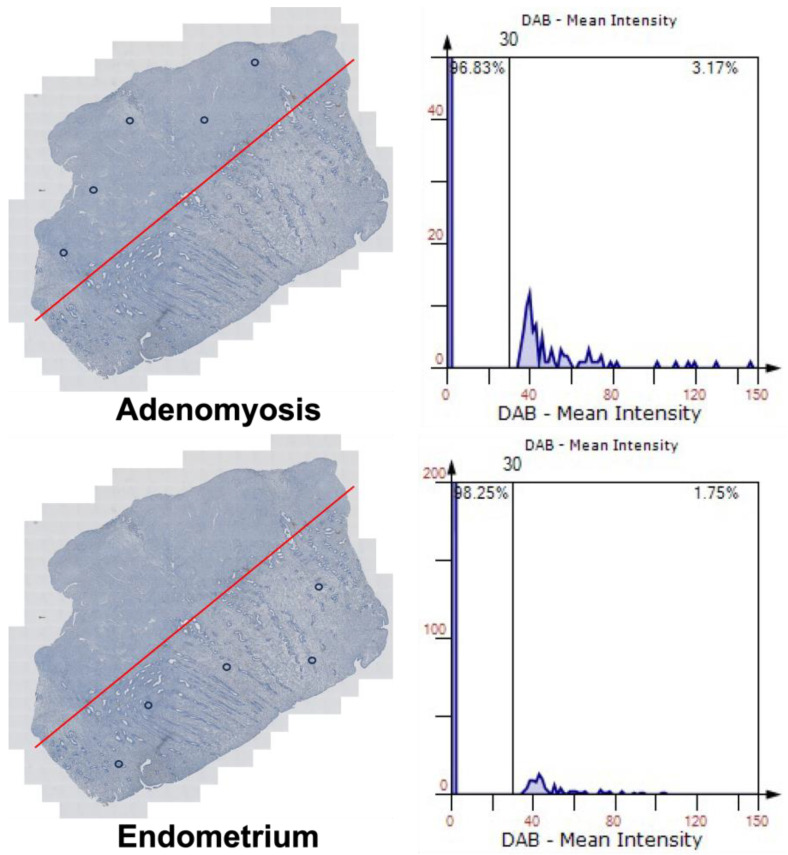
Histogram. After the paired tissues are stained by DAB, 5 areas are randomly circled at both the eutopic endometrium and adenomyosis positions. In both paired tissues, the histograms show the density information for H&E and DAB stain separately. DAB: 3,3-diaminobenzidine; H&E: hematoxylin and eosin.

We used 3,3-diaminobenzidine (DAB) as the chromogen for immunohistochemical detection of IL-17A in EMI. Figure 2 shows an example of paired samples from one patient and provides qualitative data on IL-17A expressions within the tissue samples. After the tissues were stained by immunohistochemistry, 5 areas were randomly circled at both the eutopic endometrium and ectopic endometrium (adenomyosis) positions. Histograms are well suited to visualize the relation between two tissues. We used the intensity of the HE staining as the benchmark to compare the intensity of the DAB staining between the adenomyosis and eutopic endometrium. The result shows that the mean intensity of DAB stain is higher in adenomyosis (3.17%) than in eutopic endometrium (1.75%), demonstrating IL-17A is expressed higher in adenomyosis than in eutopic endometrium.

**Figure 3:**
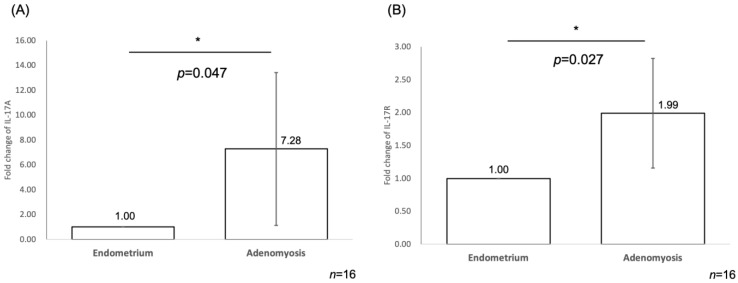
Bar graph presentation of the fold change of IL-17A (**A**) and IL-17R (**B**) transcript quantified by real-time RT-PCR. Data presented are mean ± standard deviation. (**A**) IL-17A levels are significantly elevated in the adenomyosis group compared to the eutopic endometrium group (fold change = 7.28, *p* = 0.047). (**B**) IL-17R levels are significantly higher in the adenomyosis group compared to the eutopic endometrium group (fold change = 1.99, *p* = 0.027). * *p* < 0.05. RT-PCR: reverse transcription polymerase chain reaction.

In Figure 3, we demonstrated the comparison of the fold change levels of IL-17A and IL-17R between the eutopic endometrium and corresponding ectopic endometrium (adenomyosis) group in 16 patients. Quantitative real-time reverse transcription polymerase chain reaction (RT-PCR) in paired samples was performed. The fold change of IL-17A is significantly higher in the adenomyosis group compared to the eutopic endometrium group (fold change = 7.28, *p* = 0.047, *n* = 16, Figure 3A). Similarly, the fold change of IL-17R is significantly higher in the adenomyosis group in comparison to the eutopic endometrium group (fold change = 1.99, *p* = 0.027, *n* = 16, Figure 3B).

## 3. Discussion

The endometrium showcases a sophisticated interplay among a network of cells, manifesting as a meticulously orchestrated phase encompassing proliferation, differentiation, and menstrual shedding [29,30]. The synthesis of cytokines, the expression of cytokine receptors, and the modulation of endometrial functions by these factors underscore the pivotal role of cytokines in operating at endocrine, paracrine, and autocrine levels within the endometrium. Increasingly, there is compelling evidence pointing to the participation of immune cells and inflammatory mediators in the development of endometriosis [31]. Additionally, Hirata et al. progressively reported that endometriotic stromal cells expressed IL-17RA and IL-17RC [18,32]. In 2008, this group was the first to identify IL-17A-positive cells in endometriotic tissues and Th17 cells in mononuclear cells from peritoneal fluid [18]. They also showed the expression of IL-17F in mononuclear cells from endometriotic lesions [32]. To investigate the role of the IL-17 system in adenomyosis, in our study, IL-17A and IL-17R were identified in eutopic endometrium and adenomyosis, and the fold change levels of IL-17A and IL-17R were both significantly higher in adenomyosis than in eutopic endometrium, implicating that the IL-17 system functions as a local immunomodulatory factor in adenomyosis.

In endometriosis patients, various active substances, including cytokines, growth factors, hormones, and oxidative stress parameters, have been identified at different stages of the disease [33]. An analysis revealed that the primary contributors to inflammation in endometriotic women were cytokines produced by macrophages [34]. IL-17A has been noted to function as a chemotactic agent for macrophages via its receptor, IL-17RA, and can also trigger M2 macrophage polarization in endometriotic patients [35]. Additionally, the researchers suggest that IL-17A plays a role in recruiting macrophages [36], and M2 macrophages have indeed been demonstrated to facilitate extracellular matrix remodeling and neovascularization, closely linked to the development of endometriosis [37,38,39]. The heightened production of tumor necrosis factor, triggered by activated macrophages, along with the effect of IL-17 on endometrial cells, hastens the formation of endometriosis, often leading to unexplained pelvic pain and infertility. Moreover, recent research indicated that increased IL-17 levels in PF from patients with endometriosis-associated infertility might stimulate peritoneal macrophages to produce nitric oxide synthase 2 (NOS2) and nitric oxide (NO), which can have detrimental effects on both men’s and women’s reproductive systems [40]. Therefore, elevated levels of IL-17 are associated with infertility in endometriosis.

As the research delves deeper into cytokines and endometriosis, there is increased reporting and wider confirmation of elevated IL-17 levels in endometriosis [36,41]. Zhang and colleagues were the first to demonstrate elevated IL-17 levels in the PF of endometriotic individuals. Furthermore, other studies have shown heightened levels of IL-17A in both the PF and plasma of endometriotic patients compared to controls, along with the production of IL-17A by lesions of endometriosis [36,41,42]. In conclusion, it is apparent that in women with endometriosis, the IL-17 level commonly rises, often observed in both blood and PF samples.

Histologically, adenomyosis is characterized by ectopic endometrial tissue within the myometrium. Despite extensive research, its underlying causes remain unclear, with many aspects of its development still unknown. One identified mechanism contributing to adenomyosis is the heightened ability of certain endometrial cells to invade the myometrial tissue. A hypothesis called EMI disruption (EMID) is also proposed to account for adenomyosis resulting from iatrogenic trauma to EMI [43]. Additionally, both local and systemic immune responses have been linked to the onset and maintenance of the condition. Immune system involvement coincides with hormonal irregularities and activation of the epithelial-to-mesenchymal transition pathway, facilitating the migration of endometrial cells [44]. Furthermore, intramural lesions have been observed to show increased expression of COX-2 and lipoxygenase-5, which correlates with local IL-6 and IL-8 levels. This provides support for the inflammatory hypothesis of adenomyosis pathogenesis [45]. Another study has found that tumor necrosis factor-α and vascular endothelial growth factor (VEGF) can stimulate the release of the C-X-C motif chemokine ligand 1 (CXCL1) in endometrial epithelial cells derived from the endometrium of patients with adenomyosis. This effect is enhanced with VEGF and involves several pathways, including nuclear factor kappa B (NF-κB) activation and inhibitor of nuclear factor kappa B (IκB) phosphorylation. Consequently, CXCL1 attracts migration of vascular endothelial cells, promoting local neovascularization and contributing to the progression of adenomyosis [46].

In previous studies, interleukin-18 (IL-18) was found to be possibly related to the pathologic process of adenomyosis [47,48]. Therefore, exploring the IL-17 system may offer novel perspectives on the pathogenesis and therapy of adenomyosis. In a prior study, they concluded that regulated by cytokines and estrogen, IL-17 secretion increases, either stemming from Th17 differentiation or stromal cells of endometrium. IL-17 then enhances the development, proliferation, and ectopic invasion, promoting immune evasion and endometriotic development by inducing and recruiting the M2 macrophage differentiation [35]. In conjunction with our results, the IL-17 system might participate in the formation of ectopic endometriotic lesions. IL-17 is also recognized as a key inflammatory cytokine, primarily responsible for activating tissue responses and directing a neutrophil-driven immune defense. Given its involvement in several chronic inflammatory diseases, it has become a significant target for current biologics and ongoing drug development efforts. Therapeutics targeting IL-17 were developed shortly after groundbreaking studies highlighted its crucial role in several inflammatory disorders [49]. The most extensively studied application is in the treatment of psoriasis, and IL-17A neutralization has been found to be an effective treatment for most psoriasis patients [49]. Based on these findings, the IL-17 system may be a potential biomarker for the diagnosis and therapy of adenomyosis.

Nevertheless, this study also has several limitations. Methodologically, this study is disadvantaged by an inability to completely control selection and confounding biases. Despite being the first published study reporting on expression of the IL-17 system in adenomyosis, the number of patients with adenomyosis was small. Therefore, this population is not generally representative. Furthermore, the study design limited our ability to infer causality between the IL-17 system and adenomyosis.

## 4. Materials and Methods

### 4.1. Ethical Disclosure and Tissue Sampling Methodology

**Figure 4:**
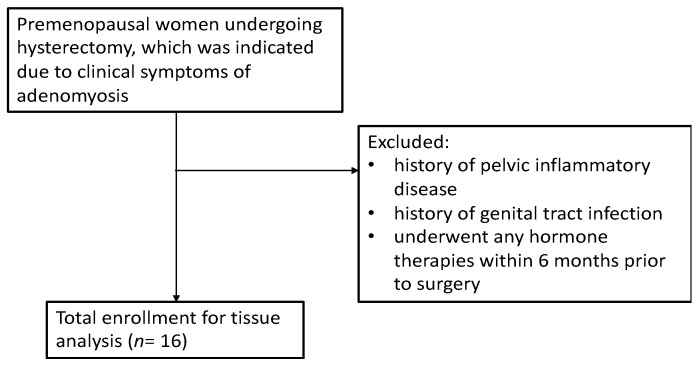
Flow chart of study subjects.

Approval for our research was granted by the Institutional Review Board of Chang Gung Memorial Hospital (IRB#201700139B0; 14 February 2017). Prior to enrollment, all participants submitted written informed consent. Finally, we enrolled 16 premenopausal women with a mean age of 42.4 ± 6.9 years and a mean body mass index (BMI) of 24.8 ± 6.2 kg/m^2^; 12 of the 16 participants (75%) had given birth at least once, and 4 of them (25%) had a history of uterine fibroids Paired tissues of their matched eutopic and ectopic endometrium (adenomyoma) were obtained from 16 participants undergoing hysterectomy. Hysterectomy was indicated due to clinical symptoms related to adenomyosis, including hypermenorrhea and dysmenorrhea. The exclusion criteria were (1) subjects with a past history of pelvic inflammatory disease; (2) subjects with a history of genital tract infection; and (3) subjects who had undergone any hormone therapies within 6 months prior to surgery (Figure 4). Biopsies were conducted by a skilled gynecologist at Linkou Chang Gung Memorial Hospital. The tissue samples were separated from the central part of the ectopic endometrium, and the length between the edge of the tissue sample and the myometrium is at least 1 cm. Histological documentation was conducted on the excised tissue samples to confirm the presence of adenomyosis foci, characterized by active endometrial glands and stroma surrounded by hypertrophic myometrium. The tissue samples used for this study were histologically proven as either proliferative (9 participants) or secretory phase (7 participants) endometrium. Sections of EMI were collected for immunohistochemistry.

### 4.2. Treatments for Specimens

Fresh tissue specimens were split into two portions. One portion was fixed in 4% formaldehyde, embedded in an optimal cutting temperature compound (OCT; Shakura Finetek, Torrance Inc., Torrance, CA, USA), and frozen in liquid nitrogen until sectioning. The other portion was immediately stored at −80 °C until RNA extraction. To study mRNA expression of the IL-17 system, total RNA was extracted using RNAzol reagent (Tel-test, Inc., Friendswood, TX, USA). RNA concentration was quantified by measuring the optical density using a Spectronic 601 spectrophotometer (Milton Roy Co., Rochester, New York, NY, USA). For immunohistochemistry staining, frozen eutopic endometrium with attached myometrium tissue was sectioned into 12 serial sections (6 μm each) from each sample. The first and last slides were stained with hematoxylin and eosin (H&E) for pathological confirmation, while the subsequent slides were stained for the IL-17 system and examined using a Nikon Microphot-FXA microscope (Nikon Instruments, Garden City, New York, NY, USA).

### 4.3. Immunohistochemistry Study of IL-17A in EMI

The presence of IL-17A in human EMI was demonstrated by the immunohistochemistry. Frozen sections of EMI were cut at 8 μm, fixed on 4% paraformaldehyde (FERAK) for 15 min, washed with 0.05% Tween 20/phosphate-buffered saline (PBS-T) for 10 min with shaking, and then incubated with 3% H_2_O_2_ (DAKO) in phosphate-buffered saline (PBS) for 10 min to eliminate endogenous peroxidase activity. Human monoclonal IL-17A antibody (Catalog No. ab189377, Abcam, Cambridge, UK) as the primary antibody was applicated and diluted 1:10 in PBS-bovine serum albumin (BSA) overnight at 4 °C. The negative control was incubated in PBS-BSA only. The EnVision kit facilitated immunohistochemical identification of IL-17A with horseradish peroxidase (HRP) (Dako, Glostrup, Denmark) system as the linker and liquid DAB as the chromogen. Positive antibody reactions were indicated by the presence of brown granules. All sections underwent counterstaining with Mayer hematoxylin, dehydration, and mounting. We used the technology “Tissomics Analyzer (TissueGnostics TissueFAXS Plus^TM^)” for quantifying the IL-17A immunohistochemical positive signal. There is only one patient in this experimental arm.

### 4.4. Immunohistochemistry Study of IL-17A and IL-17R in Paired Tissues of EMI

Tissues were fixed with methanol: acetone (1:1) (Mallinckrodt Pharmaceuticals, St. Louis, MO, USA) and resuspended in PBS (Sigma Chemical Co., St. Louis, MO, USA) until processed for immunohistochemistry. After washing with PBS, endogenous peroxidases were blocked with 1% H_2_O_2_ in 96% methanol, and non-specific binding was blocked with 5% non-fat milk in PBS at room temperature. After rinsing with 0.05% Tween-20 (Sigma-Aldrich, Burlington, MA, USA) in PBS, the slides were incubated with monoclonal mouse anti-human antibodies for IL-17A and IL-17 receptors, as the primary antibodies (IL-17A Catalog No. ab189377, IL-17RA Catalog No. ab178568, Abcam, Cambridge, UK) at a concentration of 20 μg/mL. The primary antibody omission was used as a negative control. After rinsing with PBS-T, the slides were incubated with a secondary antibody, anti-human IgG biotin conjugate (150 μg/mL) (Vector Laboratories Inc., Newark, CA, USA). The slides were incubated with Vectastatin ABC Kit (Vector Laboratories) reagent for 60 min at room temperature. Immunoreactive products were visualized by incubating the wells with the substrate solution in 0.1M Tris-hydrogen chloride (HCl) buffer (pH 8.2), blocking alkaline phosphatase activity with levamisole. Cells were counterstained with 25% hematoxylin. Positive staining by the primary antibody was indicated by the appearance of pink-colored cells under an optical microscope.

### 4.5. Quantitative Study for IL-17 mRNA Expression Using Real-Time RT-PCR

Total RNA from endometrial tissues, including both the proliferative and secretory phases, was isolated using TRIzol reagent (Invitrogen, Carlsbad, CA, USA). The tissues were placed in 1.5 mL centrifuge tubes containing 1 mL TRIzol. The tissues were homogenized using scissors until fully dissociated. Next, 200 μL of chloroform was added to each tube, which was then shaken for 15 s. After a 5 min incubation at room temperature, the tubes were centrifuged at 12,000 rpm for 15 min at 4 °C. The colorless upper aqueous phase (about 500–600 μL) was carefully removed, avoiding impurities, and transferred to a clean 1.5 mL centrifuge tube. Each tube containing the aqueous phase received 500 μL of fresh isopropanol, was mixed, and then centrifuged again at 12,000 rpm for 15 min at 4 °C. The supernatant was removed, and the RNA pellet was washed twice with 1 mL of 75% ethanol. The RNA pellet was then air-dried for 10 min at room temperature. The RNA samples were resuspended in 30–40 μL of DEPC-treated H_2_O. Finally, the RNA concentration was determined using a spectrophotometer.

Specific sequences of human oligonucleotide primers for detecting IL-17A and IL-17R were obtained from Thermo Fisher Scientific Inc. (Waltham, MA, USA) and used for PCR amplification. The IL-17 receptor subtype that we studied was IL-17RA. The Taqman^®^ gene expression assay ID for IL-17A is Hs00174383_m1 and for IL-17RA is Hs01064648_m1. Total RNA was reverse transcribed in a 20 μL volume using the SuperScript III First-Strand Synthesis SuperMix Kit (Invitrogen). The reaction was carried out by mixing the following components in a 0.5 mL PCR tube on ice, including 1–2 μg of total RNA, 1 μL of annealing buffer, and 2.5 μM oligo (dT)20 to a final concentration. The mixture was incubated at 65 °C for 5 min and then immediately placed on ice for at least 1 min. Next, 10 μL of 2X First-Strand Reaction Mix and 2 μL of SuperScript Enzyme Mix were added to the mixture. The reaction was incubated at 53 °C for 50 min and terminated at 85 °C for 5 min. The resulting cDNA was stored at −20 °C.

DNA amplification and data collection were carried out using the LighCycler System. All reactions were performed with the QuantiTect SYBR Green PCR Kit (Qiagen, Germantown, MD, USA). The reaction components comprised 1X QuantiTect SYBR Green PCR Master Mix, with a final concentration of 0.5 μM for each primer. The reactions were cycled with an initial activation step of 15 min at 95 °C, followed by 40 cycles of 15 s at 94 °C, 30 s at 60 °C, and 20 s at 72 °C. The raw data were analyzed with the Light Cycler software version 4.0. The quantitative amount of IL-17A and IL-17R was determined.

### 4.6. IL-17 mRNA Expression Determined by Quantitative RT-PCR (qRT-PCR)

Gene expression levels of IL-17A and IL-17R in paired uterine samples were assessed through quantitative real-time RT-PCR, utilizing the TaqMan methodology with the Applied Biosystems 7900 HT Real-time PCR System. PCR reactions were conducted according to the manufacturer’s guidelines, reaching a final volume of 25 μL, which comprised 12.5 μL of 2X TaqMan Universal PCR Master Mix (Life Technologies^®^, Foster City, CA, USA), 1.25 μL TaqMan assay (20×), 1 μL of sample cDNA, and 10.25 μL of RNAse-free water. The levels of IL-17A and IL-17R mRNA in adenomyosis were contrasted with those in the eutopic endometrium from the same patient. Comparative gene transcript amounts were calculated using the delta-delta Ct method [50].

### 4.7. Data Interpretation

The data are presented as the mean ± standard deviation. Data analysis was conducted using SPSS 22.0 (SPSS Inc., Chicago, IL, USA), with statistical significance set at a *p*-value of <0.05.

## 5. Conclusions

In conclusion, to our knowledge, this is the first study to compare the expression of the IL-17 system between eutopic endometrium and adenomyosis in women with adenomyosis. We found that expression of the IL-17 system is significantly higher in adenomyosis than in corresponding endometrium at the level of EMI. This study demonstrated that the IL-17 system may play a role in adenomyosis. Further investigation is required to accurately determine the role of the IL-17 system in the pathogenesis and therapy of adenomyosis.

## Data Availability

Data are available upon request.
